# Genome-Wide Association Mapping of Anthracnose (*Colletotrichum sublineolum*) Resistance in NPGS Ethiopian Sorghum Germplasm

**DOI:** 10.1534/g3.119.400350

**Published:** 2019-07-13

**Authors:** Hugo E. Cuevas, Louis K. Prom, Clara M. Cruet-Burgos

**Affiliations:** * USDA-ARS, Tropical Agriculture Research Station, Mayaguez, Puerto Rico 00680; † USDA-ARS, Southern Plains Agriculture Research Center, College Station, Texas 77845, and; ‡ Department of Biology, University of Puerto Rico, Mayaguez, Puerto Rico, 00680

**Keywords:** anthracnose, GWAS, Ethiopia, population structure, sorghum, tropical germplasm

## Abstract

The National Plant Germplasm System (NPGS) Ethiopian sorghum [*Sorghum bicolor* (L.) Moench] collection of the United States is an important genetic resource for sorghum improvement. Anthracnose (*Colletotrichum sublineolum*) is one of the most harmful fungal diseases in humid sorghum production regions. Although multiple resistance sources have been identified in temperate-adapted germplasm in the Sorghum Association Panel (SAP), these resistance loci explain a limited portion of the total variation, and sources of resistance from tropical germplasm are not available for breeding programs at temperate regions. Using a core set of 335 previously genotyped NPGS Ethiopian accessions, we identified 169 accessions resistant to anthracnose. To identify resistance loci, we merged the genotypic and anthracnose response data for both NPGS Ethiopian germplasm and the SAP and performed genome-wide association scans using 219,037 single nucleotide polymorphisms and 617 accessions. The integrated data set enabled the detection of a locus on chromosome 9 present in the SAP at a low frequency. The locus explains a limited portion of the observed phenotypic variation (*r^2^* = 0.31), suggesting the presence of other resistance loci. The locus in chromosome 9 was constituted by three R genes clustered within a 47-kb region. The presence of multiple sources of resistance in NPGS Ethiopian germplasm and SAP requires the inclusion of other resistance response evaluation that could revealed others low frequency resistance alleles in the panel.

Sorghum [*Sorghum bicolor* (L.) Moench] is the fifth most important cereal in the world and is grown for animal and human consumption (FAOSTAT 2017). The crop originated in the dry northeast region of Africa and has undergone significant diversification, leading to the formation of five distinct races (Bicolor, Caudatum, Durra, Kafir, and Guinea). These races are associated with particular environments, resistance to biotic and abiotic stress (Smith and Frederiksen 2000), and are differentiated by their inflorescence type (Snowden 1936). In 2014, the United States produced 13% of world sorghum crop, mainly in dryland farming regions in the center of the country (*i.e.*, Kansas and Texas) (USDA 2017). Today, the crop is expanding rapidly to the warm and humid Southeastern United States and Caribbean, where the long growing season makes sorghum attractive as a biofuel feedstock. This sustained expansion of sorghum production to new environmental niches requires overcoming new pathogens and pests.

Anthracnose (*Colletotrichum sublineolum* P. Henn., in Kabat and Bubák) is becoming a major disease of sorghum in warm and humid sorghum production regions worldwide. The disease appears as leaf blight, stalk rot, and head blight, but the most common and severe infection occurs on the leaf, causing yield losses of up to 50% (Thakur and Mathur 2000). The infection occurs from the 3- to 4-leaf stage to mature plants; however, highly susceptible cultivars may not reach seed maturity. While it is possible to minimize the impact of anthracnose by chemical control (Thakur and Mathur 2000), the incorporation of resistance genes is the most cost-effective and environmentally benign approach.

The field population dynamics and genetic diversity of *C. sublineolum* are not well understood (Prom *et al.* 2012). The pathogen population is highly genetically diverse, making it difficult to obtain a widespread or durable source of resistance. Nevertheless, several recent studies have found resistance loci that are effective across multiple pathotypes or environments (Cuevas *et al.* 2014a; Patil *et al.* 2017; Cuevas *et al.* 2018a). However, the continued use of these limited sources of resistance in the United States coupled with the rapid evolution of fungal pathogens could lead to the collapse of resistance genes (Mcdonald and Linde 2002). Other sources of resistance have been identified in tropical (Erpelding and Prom 2004; Erpelding 2011; Erpelding 2012; Cuevas *et al.* 2014b; Cuevas *et al.* 2016; Cuevas *et al.* 2018b) and temperate-adapted germplasm (Prom *et al.* 2016; Cuevas *et al.* 2018a). However, inheritance studies are lacking for most of these resistance sources, limiting the identification of new resistance loci for temporal deployment strategies to increase the durability of resistance sources.

A family-based approach for quantitative trait locus (QTL) mapping using the resistant temperate-adapted lines SC112-14, SC414-12E, and SC748-5 demonstrated three different genomic regions close to the distal region of the long arm of chromosome 5 were associated with anthracnose resistance (Ramasamy *et al.* 2009; Cuevas *et al.* 2014a; Burrell *et al.* 2015; Patil *et al.* 2017) in mainland United States and Puerto Rico. Using resistant sorghum lines BK-7 and SC155-14E in two mapping populations, a major anthracnose resistance locus was mapped to the telomeric region of the short arm of chromosome 9 (Felderhoff *et al.* 2016; Patil *et al.* 2017), but it is unclear if both lines contain the same resistance gene. Molecular markers linked to these QTL are valuable genomic tools for breeding programs using similar germplasm, but this approach is not useful for screening germplasm with a large number of recombination events between markers and resistance alleles may have taken place (*i.e.*, the presence of linkage equilibrium). Therefore, the identification of causal resistance genes is necessary for mining tropical sorghum germplasm to identify new resistant alleles.

The Sorghum Association Panel (SAP) was assembled to capture the majority of genetic diversity present in sorghum breeding programs in the U.S. (Casa *et al.* 2008) and was genotyped (Morris *et al.* 2013a) to establish a genomic resource for the genetic dissection of economically important traits. Among 335 accessions in the SAP, 75 highly genetically diverse accessions are resistant to anthracnose (Cuevas *et al.* 2018a). A genome-wide association analysis identified three loci within the distal region of chromosome 5 that might be associated with resistance in SC112-14, SC414-12E, and SC748-5. Nevertheless, these three loci only explain 56% of the observed phenotypic variation, implying that other resistance loci are present at low frequencies (<0.05) or are masked by overcorrection for population structure. In this regard, the United States National Plant Germplasm System (NPGS) sorghum collection could be strategically selected, genotyped, and integrated into the SAP to increase the frequency of resistant alleles and to identify loci not present in the SAP.

The NPGS holds the largest sorghum collection in the world, with more than 44,000 accessions from 114 countries. Owing to the large size of this collection and accessibility of genomic resources in sorghum, it is imperative to identify the most valuable germplasm for breeding programs based on genotypic profiles or economically important alleles for introgression into elite lines. A core set of 376 accessions from the NPGS Ethiopian germplasm collection includes 148,476 single nucleotide polymorphisms (SNPs) and comprises adequate genetic diversity and population structure for genome-wide association studies (GWAS) (Cuevas *et al.* 2017). Thus, we combined the diversity present in the SAP and the core set of NPGS Ethiopian germplasm with preexisting genomic resources for both germplasm (Morris *et al.* 2013a; Cuevas *et al.* 2017) to 1) identify new sources of anthracnose resistance in Ethiopian germplasm, 2) uncover anthracnose resistance loci in the SAP, and 3) demonstrate the effectiveness of integrating NPGS tropical germplasm and the SAP to identify new agronomically important genes/alleles not present in temperate-adapted breeding germplasm.

## Materials and Methods

### Germplasm and field experiment

A total of 335 accessions from a core set of the NPGS Ethiopian germplasm collection (Cuevas *et al.* 2017) were evaluated for anthracnose resistance (Supplemental Table S1). The core set and susceptible [BTx623, RTx430, and PI609251; Prom *et al.* (2012)] and resistant [SC112-14; Cuevas *et al.* (2014a)] controls were planted in research farms of the USDA-ARS Tropical Agriculture Research Station at Isabela (18.471569, -67.043472) and Mayaguez (18.211536, -67.135562), PR for 2 consecutive years (November 2013 and October 2014). At both locations, a completely randomized design was used, with plots measuring 1.8 m in length with 0.9 m between rows. Plants were maintained using standard management practices, weeds controlled with mechanical tillage and hand hoeing, and aerial watering (Raingun Irrigation) was supplied in the absence of rainfall. One hundred fifty-two accessions that were resistant at both locations were evaluated for 2 additional years (November 2015 and February 2018) together with referenced checks at Isabela, PR, in a randomized complete block design consisting of two blocks with plots of 1.8 m length with 0.9 m between rows.

### Anthracnose response

The inoculation and disease assessment methods were similar to those described by Prom *et al.* (2009). Two fungal cultures were prepared with different isolates of *C. sublineolum* representing the pathotypes present at the Isabela and Mayaguez research farms. The five pathotypes of Isabela were previously characterized (Prom *et al.* 2012), whereas the three pathotypes from Mayaguez have not been characterized. These isolates were applied to autoclaved sorghum seeds and placed into the sorghum leaf whorls 30 - 40 days after planting each experiment (*i.e.*, one day inoculation per experiment). Disease assessment was performed before harvesting using a scale of 1–5 as follows: 1 = no symptoms or chlorotic flecks on leaves; 2 = hypersensitive reaction on inoculated leaves but no acervuli in the center; 3 = infected bottom leaves with acervuli; 4 = necrotic lesions with acervuli on bottom leaves and spreading to middle leaves; and 5 = most leaves are dead due to infection, including infection on the flag leaf.

### Genotyping-by-Sequencing (GBS)

Genotype information for this study is a community resource generated for the NPGS Ethiopian core set (Cuevas *et al.* 2017) available at National Center for Biotechnology Information (NCBI) Sequence Read Archive (SRA) (SRP159248; Supplementary Table 1). This sequence data were combined with the SAP (Morris *et al.* 2013b) sequence information available at NCBI-SRA [(SRX1085248, SRX1085243, SRX1058235, SRX1058234, SRX1058233, SRX1085230, and SRX1085228) and another sequenced line (D0D0BACXX_6) obtained from Elizabeth Cooper (*Clemson University*, *South Carolina*, *USA*)]. Both sets were combined to call SNP genotypes using TASSEL 5 GBS v2 Pipeline (Glaubitz *et al.* 2014) and the most recent version of the BTx623 sorghum genome (version 3.1; www.phytozome.net, accessed Feb. 15, 2018). Raw genotypes (319,423 SNPs) were filtered according to minor allele frequencies (MAFs) (>0.01) and missing data (<0.25). After filtering, 224,216 SNPs were retained and missing data were imputed using Beagle 4.1 with a probability call of >0.80. The imputed genotype data set was filtered for MAF (>0.05) and missing data (<0.80) to retain 219,037 SNPs for association studies.

### Cluster analysis

The NPGS Ethiopia and SAP genotypes was pruned to 23,938 unlinked SNPs (*r^2^* < 0.10) using PLINK (Purcell *et al.* 2007). Pairwise genetic distances among a subset of 207 accessions that averaged an anthracnose infection <2.0 were calculated based on identical-by-state (IBS) as implemented in TASSEL 5.2. The resulting matrix was subject to clustering analysis using neighbor-joining and visualized using the Interactive Tree of Life (Letunic and Bork 2011).

### Phenotype Analysis

The anthracnose resistance responses of NPGS Ethiopia collection was combined with the previous study of the SAP (Cuevas *et al.* 2018a) conducted at same locations. The eight-experiment data (SAP and Ethiopia) were subject to analysis of variance using the *Proc mixed covtest* method *type 3* procedure of SAS (SAS Institute, Cary, NC). The location and accessions were considered fixed and blocks were treated as random effects. Anthracnose resistance response were estimated based on least square means and compared using Tukey-Kramar honest significant different test.

### Association Analysis

Association analyses were completed for: 1) NPGS Ethiopia collections and 2) the combined data of NPGS Ethiopia collection and SAP. The enriched compressed mixed linear model [ECMLM; (Li *et al.* 2014)] was implemented to analyze quantitative data using the Genome Association and Prediction Integrated Tool (GAPIT) in R (Lipka *et al.* 2012). For the ECMLM, the first three principal component (**Q**) and the kinship matrix (**K**) were included to control for population structure and family relatedness. Log quantile-quantile (QQ) *p*-value plots were examined to determine how well ECMLM model accounted for population structure and relatedness. Manhattan and QQ plots were visualized using the R package qqman (Turner 2014). The empirical significance thresholds for ECMLM using the NPGS Ethiopian core set [-log_10_ (*p*-value) = 5.89] and its combination with SAP [-log_10_ (*p*-value) = 6.08] were calculated with 1000 permutations for an experiment-wise error rate of *P* = 0.05.

### SNP validation

The gene candidates identified by GWAS were also partially sequenced in a subset of 96 accessions (70 and 26 from Ethiopia and SAP, respectively) via BigDye terminator chemistry (SeqWright Genomic Services, Houston, TX, USA) to confirm SNPs for further marker development. The primers were designed using Primer3 [Supplemental Table S3; (Koressaar and Remm 2007)], and the sequence chromatograms were examined using SEQUENCHER (version 4.1; GENECODES).

### Data availability

The NPGS Ethiopian core set sequence data are available at NCBI-SRA (SRP159248). Table S1 contains the list of NPGS Ethiopian core set with its SRA number. Table S2 contains the anthracnose response data. Table S3 contains the genome coordinates for the putative single nucleotide polymorphism or gene variation among three candidate genes. Supplemental material available at Figshare: https://doi.org/10.25387/g3.8127200.

## Results

### Anthracnose Resistance in NPGS Ethiopian Germplasm

The NPGS Ethiopian core set had a mean quantitative averaged of 2.52 (on a 1–5 scale). A total of 68 accessions showed different anthracnose resistance responses among locations or years (*i.e.*, accessions were resistant at one location or year but susceptible at another), whereas 169 averaged < 2.0 across all locations and years. Only 5% of the resistant accessions identified at both locations were reclassified as susceptible after two additional years of evaluation. Most of the resistant accessions from the SAP identified in Puerto Rico are also resistant to pathotypes from other locations (Cuevas *et al.* 2018a). Therefore, we expected the majority of these 169 resistant accessions to be resistant to other pathotypes.

The population structure of the NPGS Ethiopian core set was associated with the observe anthracnose resistant response variation. Based on the previous NPGS Ethiopia population structure analysis the Ethiopian germplasm could be divided into eleven populations (Cuevas *et al.* 2017) that differ in anthracnose resistant response (Table 1). We observed six populations averaged low anthracnose infection (<2.42; Population 5, 6, 7, 9, 10, and 11) and one population highly susceptible (4.49; Population 1). The high genetic relatedness suggests that resistant accessions in Ethiopian populations share the same resistance mechanism (*i.e.*, identical by descent). Certainly, evaluate the most resistant populations for other agronomic important traits increase the likelihood to identify accessions that combine anthracnose resistance and superior agronomic traits.

**Table 1:** Least square means of anthracnose resistance response in a core subset of NPGS Ethiopian germplasm and SNP frequency of anthracnose resistant loci in *Sobic.009G013300*

		Ethiopia	Chr9: 1176056	
Populations*^a^*	n	Means ± SD	T*^b^*	C
1	8	4.15 ± 0.37 a	0.00	1.00
2	17	3.28 ± 0.25 ab	0.05	0.95
3	26	2.97 ± 0.21 ab	0.42	0.58
4	7	2.46 ± 0.40 ab	0.00	1.00
5	5	1.73 ± 0.43 b	0.00	1.00
6	35	2.42 ± 0.18 b	0.07	0.93
7	21	2.23 ± 0.23 b	0.80	0.20
8	6	2.63 ± 0.43 ab	0.00	1.00
9	24	1.73 ± 0.21 b	0.04	0.96
10	15	2.18 ± 0.27 b	0.38	0.62
11	8	2.16 ± 0.37 b	0.00	1.00
Admixed	151	2.64 ± 0.09 ab	0.18	0.82

a Population structure according to Cuevas *et al.* (2017).

b Anthracnose-resistant allele.

**Figure 1:**
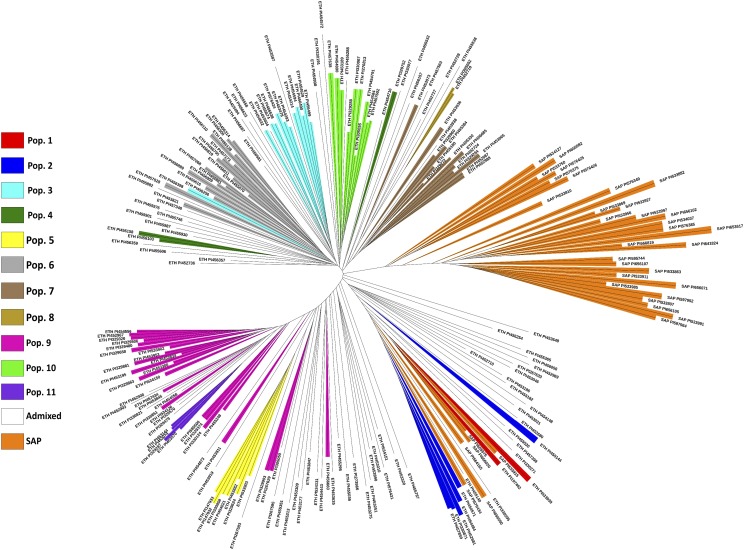
Unrooted neighbor-joining tree of 209 anthracnose resistant accessions present in the NPGS Ethiopian core set and in the Sorghum Association Panel (SAP). Colors represent the NPGS Ethiopian populations and the SAP.

The NPGS Ethiopian anthracnose-resistant germplasm is a resource that could be used to increase the genetic diversity in the SAP. We generated an unrooted neighbor-joining tree to evaluate the genetic relationships among 207 resistant accessions in NPGS Ethiopian germplasm and the SAP (Figure 1). Most of the resistant accessions in Ethiopian populations could be used to expand the genetic diversity of the SAP. The resistant accessions in populations 1, 2 and 5 clustered with 7 accessions from SAP indicating these resistance sources were probable integrated in the SAP. The introgression of early maturity and dwarfing genes in converted sorghum germplasm present in the SAP has reduced genetic variation in multiple genomic regions (Thurber *et al.* 2013). Nevertheless, we obtained similar results by generating a second unrooted neighbor-joining tree excluding chromosome 6 and a genomic region on chromosome 9 associated with early maturity (*Ma1*) and dwarfing (*dw1*), respectively.

### Genome-Wide association analysis

**Figure 2:**
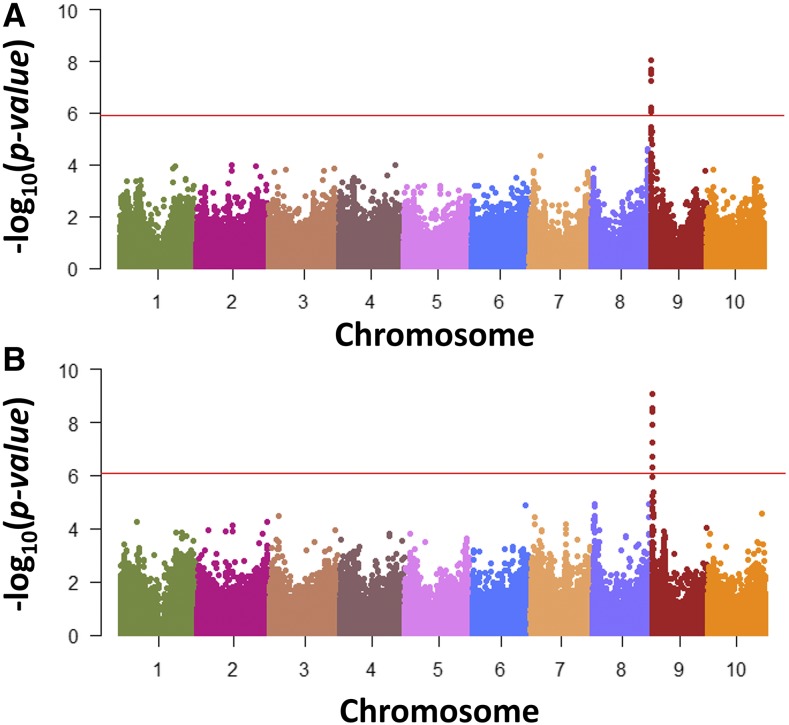
Genome-wide association analysis of anthracnose resistance using quantitative data. (a) Manhattan plots for ECMLM using Ethiopian sorghum germplasm and red horizontal line referring to an experiment-wise significance threshold of *P* < 0.05 based on 1000 permutations. (b) Manhattan plots for ECMLM using both Ethiopian sorghum germplasm and the U.S. Sorghum Association Panel and red horizontal line referring to an experiment-wise significance threshold of *P* < 0.05 based on 1000 permutations.

In a genome-wide association scan of Ethiopian germplasm we detected an association between the telomeric region of chromosome 9 [Chr.9: 1,176,056; -log(*p*-value) = 8.02] and anthracnose resistance (Figure 2). We observed the strongest association [Chr.9: 1,176,056; -log(*p*-value) = 9.07] when both Ethiopian and SAP anthracnose-resistant responses were merged and scanned using the ECMLM. The regression model including the SNP explains 0.25 and 0.31 of the variation, respectively. The genomic region on chromosome 9 has previously been associated with anthracnose resistance in the sorghum line Bk7 (Felderhoff *et al.* 2016) and SC155-14E (Patil *et al.* 2017), however, both family approaches resulted in the identification of large genomic region with multiple candidate genes. All analyses had adequate control for population structure and familial relatedness based on a visual inspection of QQ plots (Supplemental Figure S1).

### Candidate genes and allele frequency distribution

The region on chromosome 9 based on ECMLM was 78.7-kb with 8 SNPs in linkage disequilibrium (*D′* > 0.79). One SNP was located within the intronic region of the putative gene *Sobic.009G012500* (Replication Protein A 70 kD DNA Binding Subunit C-related), another was in the coding region of the putative gene *Sobic.009G012900* [Leucine-rich repeat; (LRR)], and six were in the coding region of the putative gene *Sobic.009G013300* [LRR, Coiled-coil domain (CC) containing protein lobo homolog, nucleotide binding domain (NBD), and apoptotic ATPase]. Indeed, the most prevalent R genes in plant families contain LRRs, a central nucleotide binding site, and a variable amino terminal domain that activates signaling cascades involved in the defensive response (Bent and Mackey 2007).

The frequency of the resistant allele of *Sobic.009G013300* among Ethiopian resistance accessions was 0.30, while among SAP resistance accessions was 0.12. When Ethiopian and SAP resistance accessions are merged the frequency is 0.27. Therefore, other important genes and combinations of alleles related to anthracnose resistance in both germplasms remains uncovered. Likewise, the frequency of resistant allele of *Sobic.009G013300* was associated with resistance response observe in populations 3, 7, 10 and the admixed group (Table 1). The further integration of others germplasm into the association analysis or the development of mapping populations derived from strategically selected resistance accessions present in other populations are necessary to identify other novel resistant loci.

### SNP validation

A resequencing analysis of *Sobic.009G013300* was hampered by the presence of two homologous genes (*Sobic.009G013000* and *Sobic.009G013100*) with more than 90% similarity. Thus, we only sequenced an amplicon of 699 bp for the three genes, including the most significant SNP (S9_1176056). Based on the sequence alignment, we confirmed S9_1176056 and identified 17 putative SNPs (Supplemental Table S3), which could not be linked to a single homologous gene. Therefore, they may be used for marker-assisted selection if they segregate in biparental mapping populations.

## Discussion

The genomic dissection of multiple anthracnose resistance sources in the SAP is necessary to initiate a temporal deployment system to increase resistance durability in United States breeding programs. Despite the discovery of three anthracnose resistance loci on chromosome 5 by GWAS conducted with logistic regressions, owing to the low power to detect loci at low frequencies, candidate resistance alleles have not been identified for most of the resistance accessions in SAP (Cuevas *et al.* 2018a). In this study, we integrated the SAP and NPGS Ethiopian germplasm, leading to the identification of an anthracnose-resistant allele in *Sobic.009G013300* present in the SAP at a low frequency. This locus could be associated with three resistant accessions in the SAP originally from Ethiopia (SC1014, SC1033, and SC155) and two advanced sweet sorghum cultivars (Keller and Wray). Remarkably, resistant alleles for the three resistance loci on chromosome 5 that were previously identified by a GWAS using only the SAP were absent in three accessions (SC1014, SC1033, and Wray). Although SC155 includes resistant alleles for other loci on chromosome 5 based on biparental mapping populations, the QTL on chromosome 9 controls anthracnose resistance against pathotypes from Georgia and Texas, USA (Patil *et al.* 2017). Other resistance loci present in the SAP at low frequencies are controlling the observe resistance response in SAP. Thus, the genotyping and screening of NPGS tropical germplasm for anthracnose resistance is valuable tool to uncover additional resistance alleles present in the SAP.

The NPGS Ethiopian germplasm collection may include resistant sources that are currently absent in the SAP. Resistant alleles for *Sobic.009G013300* are dispersed among six Ethiopian populations and only explained a small portion of the observe phenotypic variation (*r^2^* = 0.31). In fact, 104 Ethiopian resistance accessions do not enclose the resistance allele for *Sobic.009G013300*. Most of the accessions from the NPGS Ethiopia collection belong to the sorghum race Durra and its intermediates (GRIN 2018). We observed that the panicle shape variation among the majority of anthracnose-resistant accessions resembles that of intermediates derived from the Guinea sorghum race. In fact, most of the accessions in Ethiopian populations with high frequency of the resistance alleles (*i.e.*, populations 3, 7 and 10) belongs to Durra race. These results suggest that resistance allele for *Sobic.009G013300* could be originated within the Durra (Ethiopian) genetic background. Hence, resistance sources originally from other sorghum races might be present in the germplasms at low frequencies. Ethiopia is considered the center of origin of the crop, where four of the five sorghum races and wild forms are present (Teshome *et al.* 1997); thus this germplasm appears to include both resistance sources evolved in the region and from other African countries. For instance, resistance sources present in populations 1, 4, 5, 8 and 11 are not associated with *Sobic.009G013300*. Remarkable, seven resistance accessions from SAP (Red Kafir, RTx434, SC1494, Ex Mubi, SC79 and BTx406) are genetically related to accessions from populations 1 and 5. Thus, other resistance sources present in Ethiopian germplasm are also available to sorghum breeding programs. The anthracnose-resistant molecular mechanism requires interactions with other proteins fixed in the genetic backgrounds of particular races (Cao *et al.* 2007). Hence, resistance sources originally from other sorghum races (*e.g.*, Caudatum, Kafir, Guinea, and Bicolor) are unlikely to be detected in our analysis. Certainly, a representative subset of the 104 anthracnose-resistant accessions without resistance alleles for *Sobic.009G013300* and not related to Ethiopian populations 1, 2 and 5 could be adapted to temperate regions as new sources of resistance for breeding programs.

The low frequency of the resistance allele for *Sobic.009G013300* in NPGS Ethiopia (0.20) and SAP (<0.05) is related to the origin of both germplasms. The SAP was constituted to capture most of the genetic diversity present in U.S. breeding programs (Casa *et al.* 2008), thus, these resistance accessions were primary selected by other economic important traits (*e.g.*, yield). The high frequency of resistance alleles in three Ethiopian populations may be explained by selection pressure driven by humans and natural conditions. The further association of Ethiopian populations with adaptation to environmental regions is necessary to understand the evolution of the resistance allele.

The plant immune system has four components: (i) pathogen recognition, (ii) signal transduction, (iii) downstream defense-related gene activation, and (iv) crosstalk among signaling pathways (Helliwell and Yang 2013). The gene *Sobic.009G013300* (NBD-LRR-CC) is involved in pathogen recognition and clustered with two homologous gene. These two homologs (*Sobic.09G013000* and *Sobic.09G013100*) with high sequence similarity (>90%) are clustered within a 47-kb region. Fungal resistance genes are often clustered and display a multiallelic structure against different pathotypes or diverse pathogens, playing an important role in plant environmental adaptation (Giraud *et al.* 2010). In fact, NBD-LRR genes are under strong selection pressure and have had significant effects on sorghum evolution (Mace *et al.* 2014). It is necessary to determine the role of these homologous genes in anthracnose resistance or other fungal diseases to precisely delimit the genomic region to be introgressed in susceptible germplasm. While the cluster of genes could be backcrossed into susceptible germplasm, it has the potential to inadvertently introduce susceptibility to other diseases. As a post-invasion resistance mechanism in plants, most resistance genes are dominant; their products directly or indirectly detect specific pathogen effectors and trigger an effective defense response (Gururani *et al.* 2012).

### Conclusion

The NPGS Ethiopian collection is a genetically diverse germplasm with a high frequency of anthracnose-resistant accessions. Anthracnose resistance loci on chromosomes 9 (*Sobic.009G013300*) explain only a portion of the observed resistance in NPGS Ethiopia accessions. This resistance allele is also present in temperate-adapted germplasm at low frequency that cannot be detected by a GWAS conducted with the SAP. The integration of NPGS Ethiopia germplasm and SAP for GWAS analysis improved the statistical power to identified resistance genes present in SAP at low frequencies. Furthermore, understanding the genetic control of anthracnose resistance in NPGS tropical germplasm is imperative to ensure that new resistance sources are introgressed into temperate-adapted germplasm. The cluster of R genes associated with the locus on chromosome 9 may be associated with pathogen recognition, but further studies are needed to determine the exact roles of these homologous genes.
